# Forebrain EAAT3 Overexpression Increases Susceptibility to Amphetamine-Induced Repetitive Behaviors

**DOI:** 10.1523/ENEURO.0090-24.2024

**Published:** 2024-04-08

**Authors:** Jared M. Kopelman, Muhammad O. Chohan, Alex I. Hsu, Eric A. Yttri, Jeremy Veenstra-VanderWeele, Susanne E. Ahmari

**Affiliations:** ^1^Department of Psychiatry, Translational Neuroscience Program, University of Pittsburgh, Pittsburgh, Pennsylvania 15260; ^2^Center for the Neural Basis of Cognition, Carnegie Mellon University, Pittsburgh, Pennsylvania 15260; ^3^Department of Psychiatry, Columbia University, New York, New York 10032; ^4^New York State Psychiatric Institute, New York, New York 10032; ^5^Department of Biological Sciences, Carnegie Mellon University, Pittsburgh, Pennsylvania 15260

**Keywords:** amphetamine, basal ganglia, D1-MSN, DeepLabCut, immediate early gene, neurotransmitter transporters, preclinical model, repetitive behavior, RNAScope, striatum

## Abstract

Obsessive-compulsive disorder (OCD) is a debilitating psychiatric disorder characterized by intrusive obsessive thoughts and compulsive behaviors. Multiple studies have shown the association of polymorphisms in the *SLC1A1* gene with OCD. The most common of these OCD-associated polymorphisms increases the expression of the encoded protein, excitatory amino acid transporter 3 (EAAT3), a neuronal glutamate transporter. Previous work has shown that increased EAAT3 expression results in OCD-relevant behavioral phenotypes in rodent models. In this study, we created a novel mouse model with targeted, reversible overexpression of *Slc1a1* in forebrain neurons. The mice do not have a baseline difference in repetitive behavior but show increased hyperlocomotion following a low dose of amphetamine (3 mg/kg) and increased stereotypy following a high dose of amphetamine (8 mg/kg). We next characterized the effect of amphetamine on striatal cFos response and found that amphetamine increased cFos throughout the striatum in both control and *Slc1a1*-overexpressing (OE) mice, but *Slc1a1*-OE mice had increased cFos expression in the ventral striatum relative to controls. We used an unbiased machine classifier to robustly characterize the behavioral response to different doses of amphetamine and found a unique response to amphetamine in *Slc1a1*-OE mice, relative to controls. Lastly, we found that the differences in striatal cFos expression in *Slc1a1*-OE mice were driven by cFos expression specifically in D1 neurons, as *Slc1a1*-OE mice had increased cFos in D1 ventral medial striatal neurons, implicating this region in the exaggerated behavioral response to amphetamine in *Slc1a1*-OE mice.

## Significance Statement

Obsessive-compulsive disorder is a debilitating psychiatric disorder with inadequate treatment options. The gene *SLC1A1* has been associated with OCD in humans, and studies in rodents have shown alterations in OCD-relevant behavior and neural activity in mice with increased *Slc1a1* expression. We created a novel mouse model with reversible forebrain overexpression of *Slc1a1* and found that these mice show increased behavioral response to amphetamine. Using an unbiased machine classifier, we found differences in clusters of amphetamine-induced behaviors in *Slc1a1*-overexpressing (OE) mice. In addition, *Slc1a1*-OE mice showed increased neuronal activation in D1-expressing cells in the ventromedial striatum following amphetamine administration. These results provide information about the role of *Slc1a1* in repetitive behaviors and may contribute to novel treatments going forward.

## Introduction

Obsessive-compulsive disorder (OCD) is a debilitating neuropsychiatric disorder with a lifetime prevalence of 2–3% (Kessler et al., 2005; Ruscio et al., 2010). It is characterized by intrusive thoughts or urges known as obsessions and repetitive behaviors known as compulsions, which are often performed to relieve anxiety associated with obsessions (Pauls et al., 2014). Current standard-of-care treatments for OCD include cognitive behavioral therapy and serotonin reuptake inhibitors (SRIs). While these treatments are beneficial for many, up to 50% of OCD patients remain symptomatic (Koran, 2000; Dougherty et al., 2004), and better treatments based on the underlying neurobiology of the disorder are needed.

Neuroimaging studies of OCD have identified abnormalities of cortico-striato-thalamo-cortical circuitry in OCD patients, with hyperactivity of cortical and striatal regions (Menzies et al., 2008; Radua et al., 2010). Some studies have also reported increased glutamatergic signal in the caudate, measured by magnetic resonance spectroscopy, and increased glutamate in the cerebrospinal fluid of a subset of OCD patients (Rosenberg et al., 2000; Starck et al., 2008; Pittenger et al., 2011). Animal models have further implicated particular glutamatergic inputs from cortex to striatum in OCD-relevant behaviors (Welch et al., 2007; Corbit et al., 2019), indicating that dysfunction of these circuits may be relevant to abnormal repetitive behaviors across species.

In addition to these neuroanatomical studies, progress has been made in understanding the genetic basis of OCD, with early linkage studies implicating the chromosome 9p24 region in the pathogenesis of the disorder (Hanna et al., 2002; Willour et al., 2004). This region contains *SLC1A1*, which encodes the neuronal glutamate transporter, EAAT3, and subsequent association studies have implicated various polymorphisms of this gene in OCD (Arnold et al., 2006; Dickel et al., 2006; Stewart et al., 2007; Shugart et al., 2009; Wendland et al., 2009; Samuels et al., 2011; Veenstra-VanderWeele et al., 2012; Cai et al., 2013). The most commonly associated rs301430C allele results in increased expression of *SLC1A1* in human postmortem brains, lymphoblastoid cells, and a luciferase reporter assay (Wendland et al., 2009; Veenstra-VanderWeele et al., 2012). Despite the lack of genome-wide association (Stewart et al., 2013; Mattheisen et al., 2015), these data suggest that elevated levels of *SLC1A1*/EAAT3 may result in an increased risk of OCD in humans and may lead to OCD-relevant behaviors in model systems.

Animal studies have implicated *Slc1a1*/EAAT3 in abnormal repetitive behaviors. We recently found that mice with developmental overexpression of *Slc1a1* specifically in midbrain dopaminergic neurons showed a significant increase in amphetamine-induced repetitive behaviors, including hyperlocomotion and stereotypy (Chohan et al., 2022). A recent study by Delgado-Acevedo et al. (2019) found that overexpressing (OE) *Slc1a1* in the forebrain resulted in increased anxiety-like behaviors and repetitive behaviors, including increased grooming that was reversed by the administration of a serotonin reuptake inhibitor. Furthermore, they found abnormalities in corticostriatal synapses, including changes in NMDA receptor subunit expression and altered NMDA-dependent synaptic plasticity (Delgado-Acevedo et al., 2019). Follow-up work showed that administration of amphetamine to these mice resulted in altered grooming syntax and increased dopamine release in EAAT3-OE mice (Escobar et al., 2021). In the present study, we examined the role of EAAT3 in amphetamine-induced repetitive behavior and striatal activation. We created an inducible *Slc1a1*-OE mouse model with increased EAAT3 expression in forebrain regions and characterized the behavior of these mice, both at baseline and following amphetamine, using an unbiased machine classifier. We found that these mice showed increased amphetamine-induced behavior, including locomotion and stereotypy. We then characterized the striatal response to amphetamine in these mice. We found increased neuronal activation in D1-expressing cells in the ventromedial striatum (VMS), as measured by the immediate early gene cFos, in *Slc1a1*-OE mice.

## Materials and Methods

### Mice

All procedures were carried out in accordance with the guidelines set out by the NIH in the Guide for the Care and Use of Laboratory (National Institutes of Health, 1996) and were approved by the University of Pittsburgh or New York Psychiatric State Institute Institutional Animal Care and Use Committee. All mice were housed in cages of 3–5 mice/cage with *ad libitum* access to food and water. Mice were on a 12 h light/dark cycle, with lights on at 7:00 A.M. and lights off at 7:00 P.M., and all behavioral testing was conducted during the light cycle.

**Figure 1.**
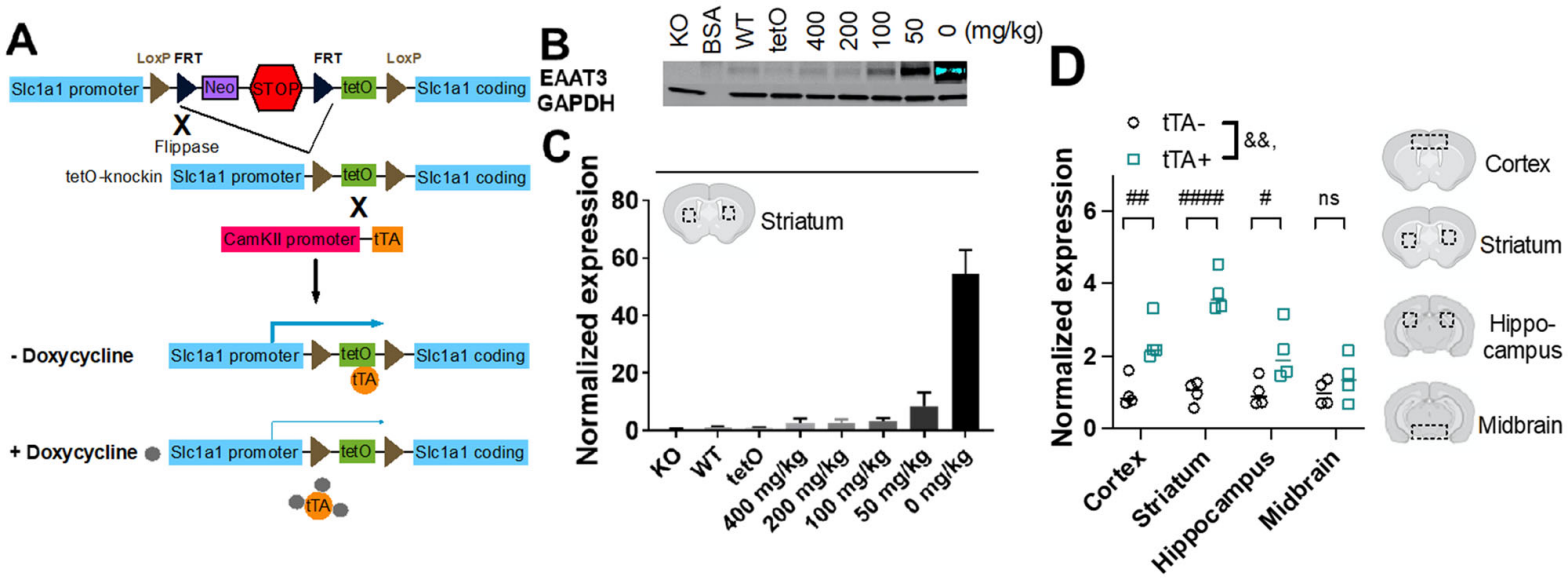
*Slc1a1*-OE mice show increased EAAT3 protein expression that is reversible with doxycycline administration. ***A***, Schematic of *Slc1a1* overexpression in *Slc1a1*-tetO/*CaMKII*-tTA mice. The neo-STOP cassette is excised by crossing with a flippase-expressing line. Crossing to *CaMKII*-tTA results in doxycycline-dependent overexpression specifically in forebrain neurons. ***B***, Representative Western blot of EAAT3 expression in the striatum of *Slc1a1*-KO, WT, *Slc1a1*-tetO, and *Slc1a1*-tetO/*CaMKII*-tTA mice receiving one of five different doses of doxycycline. ***C***, Quantification of expression in the same groups (one-way ANOVA, *F*_(7, 16)_ = 53.81, ***p < 0.001, *n* = 3 mice/group). ***D***, *Slc1a1*-OE mice fed 40 mg/kg show increased EAAT3 expression in the cortex, striatum, and hippocampus, but not in the midbrain (two-way ANOVA; genotype–region interaction, ^&&^*p *< 0.01; genotype, ****p < 0.0001; Sidak's multiple comparisons: cortex, ^##^*p *< 0.01; striatum, *^####^p < *0.0001; hippocampus, ^#^*p < *0.05; and midbrain, ^ns^*p = *not significant).

*Slc1a1*-OE mice were bred by crossing *Slc1a1*-neo-STOP-tetO mice (Zike et al., 2017) with *Pgk1*-flpo mice (Wu et al., 2009) to excise the neo-STOP cassette. Forebrain-specific overexpression was achieved by crossing these *Slc1a1*-tetO mice on a 129S6/SvEv background with *CaMKII*-tetracycline transactivator (tTA; Mayford et al., 1996) mice on a C57Bl/6 background (Fig. 1*A*), resulting in *Slc1a1*-tetO/*CaMKII*-tTA experimental mice on a mixed 129S6/SvEv;C57Bl/6 background. *Slc1a1*-OE mice were homozygous for the *Slc1a1*-tetO allele and hemizygous for the *CaMKII*-tTA transgene while tTA− control mice were homozygous for the *Slc1a1*-tetO allele only. For the doxycycline (dox) dose–response experiment, mice were fed dox at doses of 0, 50, 100, 200, or 400 mg/kg in their chow for 4 weeks prior to sacrifice (Envigo custom diet in LabDiet 5P76, ProLab IsoPro RMH 3000). For lifetime overexpression, breeding pairs were fed 50 mg/kg dox-chow, and mice were continued on this diet following weaning. For adult-specific overexpression, breeding pairs were fed 400 mg/kg dox-chow and switched to 50 mg/kg dox-chow at 8 weeks of age. For the regional expression cohorts, mice were raised on 40 mg/kg dox-chow. The dose–response and regional expression cohorts consisted of three and four mice/group, respectively. The initial lifetime overexpression cohort (baseline grooming, SKF-38393–induced grooming, and amphetamine-induced behaviors) consisted of 26 tTA + mice (14 male) and 20 tTA− mice (11 male), while the initial adult-specific overexpression cohort for these same behaviors consisted of 22 tTA + mice (10 male) and 18 tTA− mice (8 male). The second lifetime overexpression cohort (anxiety-like behaviors) consisted of 21 tTA+ mice (11 male) and 17 tTA− mice (8 male), while the second adult-specific overexpression cohort for these same behaviors consisted of 20 tTA+ mice (10 male) and 16 tTA− mice (8 male). The first cFos cohort included 16 tTA+ (7 male) and 16 tTA− mice (8 male), while the second cohort consisted of 21 tTA+ male (10 male) and 22 tTA− mice (11 male). All mice were 3–6 months at the time of testing.

### Behavioral testing

All testing was conducted between the hours of 8:00 A.M. and 6:00 P.M. The time of testing was counterbalanced by genotype and drug treatment. Mice were habituated in their home cages to the testing room for at least 20 min prior to testing. Each behavioral apparatus was washed with 70% EtOH and allowed to dry completely between mice.

### Anxiety-like behavioral testing

Open-field and light/dark behavioral testing was performed according to the procedures outlined in a previous study (Delgado-Acevedo et al. 2019). Briefly, for open-field testing, mice were placed into the center of a 41 cm × 41 cm Plexiglas open-field chamber under room lights for 30 min. Locomotor activity was scored by detecting interruptions of infrared beams by the body of the mouse; data were collected and analyzed using MotorMonitor (Kinder Scientific). The % time in the center (10 cm × 10 cm) of the chamber was recorded. For light/dark testing, the light/dark chamber consists of two equally sized chambers (24 cm × 24 cm), one enclosed and dark and one brightly lit (∼300 lux). Mice are placed in the dark side of the chamber and allowed to freely explore both sides for 10 min. Locomotor activity was scored by detecting interruptions of infrared beams by the body of the mouse, and data were collected and analyzed using MotorMonitor (Kinder Scientific). The % time spent in the light and dark sides of the chamber was recorded.

### Grooming assessment

For assessment of grooming behavior, mice were placed in a plexiglass chamber (20 cm × 20 cm) and allowed to habituate for 20 min. Behavior was then video recorded (Cannon, 720p and 60 fps) for 30 min. A trained experimenter blind to group then scored the amount of time spent grooming using a stopwatch.

### Amphetamine-induced hyperlocomotion

Mice were weighed prior to being placed into the center of a 41 cm × 41 cm Plexiglas open-field chamber for 30 min. Mice were pseudorandomized using Excel (Microsoft) to receive intraperitoneal injections of either saline or 3.0 mg/kg d-amphetamine (Sigma-Aldrich) and were placed back in the locomotor chamber for another 60 min. One week later, mice were given the other treatment in a crossover design. Locomotor activity was scored by detecting interruptions of infrared beams by the body of the mouse; data was collected and analyzed using MotorMonitor (Kinder Scientific).

### Amphetamine-induced stereotypy

Mice were weighed prior to receiving a high dose (8.0 mg/kg) of d-amphetamine via intraperitoneal injection. Mice were placed into small (20 cm × 20 cm) Plexiglas chambers and video recorded for 90 min following injection. An experimenter blind to genotype, drug treatment, and timepoint scored three 2 min time bins (Cohort I) or nine 2 min time bins (Cohort II). Stereotypy was defined as stationary head bobbing, sniffing, shuffling, or licking motion lasting at least 1 s. Interrater reliability for this behavior exceeded 0.90.

### DeepLabCut

Mice were weighed before being placed in small (20 cm × 20 cm) Plexiglas chambers for 30 min. After 30 min, mice were removed from the chamber and injected with vehicle, 3.0 mg/kg amphetamine or 8.0 mg/kg amphetamine before being placed back into the chamber. For this experiment, the behavior was video recorded (Cannon, 1,080p and 60 fps) from below the chambers. Videos were then analyzed using DeepLabCut (DLC; Mathis and Warren, 2018; Nath et al., 2018, 2019). DLC uses convolutional neural networks to estimate 3D poses. We manually labeled the location of the paws, nose, and tail base in 833 frames taken from a subset of behavioral videos. We then trained the network to predict the location of these six body parts in every other frame of the video, using a GPU (RTX 2080Ti in an Alienware R8). The training regimen was set to DLC default and trained until achieving a loss of 0.002.

### Behavioral segmentation of open-field in DLC (B-SOiD)

B-SOiD is an unsupervised learning algorithm that serves to discover and classify behaviors in an unbiased fashion (Hsu and Yttri, 2019). This algorithm segregates statistically different, subsecond rodent behaviors, using novel expectation maximization fitting of Gaussian mixture models on *t*-distributed stochastic neighbor embedding. The original features taken from dimensionally reduced classes are then used to build a multiclass support vector machine classifier that can decode actions. We trained this classifier using three 1 min videos taken from each mouse in the experiment: 1 min during habituation, 1 min during early drug response (30 min following drug injection), and 1 min during late drug response (60 min postinjection). We did this to ensure that all drug responses were represented. We then used this trained classifier to analyze all data from every mouse.

### Western blot

Western blot was performed according to Zike et al. (2017). Briefly, brains were extracted from mice after rapid decapitation and immediately frozen on an ice-cold metal platform. Sections were cut on a microtome or using a brain matrix, and the whole striatum, cortex, hippocampus, and midbrain regions were dissected and homogenized. Protein concentrations of all samples were determined by a bicinchoninic acid protein assay (Thermo Fisher Scientific). Equal amounts of protein were incubated with a Laemmli sample buffer for 5 min at room temperature (RT). Samples were analyzed by SDS/PAGE followed by Western blotting using primary rabbit polyclonal anti-EAAC1/EAAT3 antibody (1:1,000 dilution; EAAC110A, Alpha Diagnostic International), anti-GAPDH (1:1,000, Millipore), and anti-actin (1: 10,000 dilution, Abcam) antibodies and secondary anti-rabbit (GE HealthCare Life Sciences, NA934) and anti-mouse (NA931) HRP-conjugated antibodies (both 1:1,000 dilution, GE HealthCare Life Sciences). Blots were detected using Amersham ECL Prime Western Blotting Detection Reagent (RPN2232; GE HealthCare Life Sciences) and visualized via chemiluminescence using the FluorChem M System (ProteinSimple). Blots were quantified using NIH ImageJ software (version 1.53).

### Immunohistochemistry

Following the 8.0 mg/kg amphetamine challenge, mice in Cohort II were killed 50 min following behavior (140 min postinjection) and perfused with 4% paraformaldehyde and postfixed overnight at 4°C, transferred to 30% sucrose solution until they sank, frozen on dry ice, and sliced on a cryostat into 35 µm coronal sections. Sections were stored in 1× phosphate-buffered saline (PBS)/0.1% sodium azide until use. Sections were washed in Tris-buffered saline (TBS), incubated in 0.03% H_2_O_2_ for 10 min, washed again in TBS, and blocked in 3% normal goat serum before being blocked in anti-cFos primary antibody (1:1,000 dilution, MilliporeSigma) for 48 h at 4°C. Sections were washed in TBS+ (0.3% Triton X-100; Sigma-Aldrich) and incubated in anti-rabbit biotinylated secondary antibody (1:500 dilution) for 2 h, blocked in tertiary avidin-biotin complex solution (VectorLabs) for 1 h, and then stained with 3,3′-diaminobenzidine chromogen (DAB; Sigma-Aldrich) for 5 min. In between these steps, sections were washed with TBS+. Sections were mounted on glass slides, dehydrated with ethanol, coverslipped with DPX, and imaged with a light microscope. cFos-positive cells were quantified using cellSens (Olympus) by trained experimenters blinded to the treatment group.

### RNAScope

For RNAScope analysis, mice were killed 50 min following behavior (140 min postinjection). We performed RNA in situ hybridization (ISH) for Fos, Drd1, and Drd2 mRNAs as described previously (Li et al., 2015; Caprioli et al., 2016). We rapidly extracted and froze brains on dry ice. Brains were stored at −80°C until use. Brains were sliced on a cryostat at 16 µm and collected directly onto Superfrost Plus slides (Thermo Fisher Scientific). We used an RNAscope Multiplex Fluorescent Reagent Kit (Advanced Cell Diagnostics) and performed the ISH assay according to the user manual for fresh-frozen tissue. We fixed brain slices in 4% PBS for 20 min at 4°C. We rinsed the slices three times in PBS and dehydrated the slices in 50, 70, 100, and 100% ethanol. We dried the slides at RT for 10 min, and to limit the spreading of the solutions, we drew a hydrophobic barrier on slides around brain slices. We then treated the slides with protease solution (Pretreatment 4) at RT for 20 min. We then applied target probes for Fos, Drd1, and Drd2 to the slides and incubated them at 40°C for 2 h in the HybEZ oven (Advanced Cell Diagnostics). Each RNAscope target probe contains a mixture of 20 ZZ oligonucleotide probes that are bound to the target RNA, as follows: Fos-C3 probe (GenBank accession number NM_022197.2); Drd1-C1 probe (GenBank accession number NM_012546.2); and Drd2-C2 probe (GenBank accession number NM_012547.1). Next, we incubated the slides with preamplifier and amplifier probes (AMP1, 40°C for 30 min; AMP2, 40°C for 15 min; AMP3, 40°C for 30 min). We then incubated the slides with fluorescently labeled probes by selecting a specific combination of colors associated with each channel, as follows: green (Alexa Fluor 488 nm), orange (Alexa Fluor 550 nm), and far red (Alexa Fluor 647 nm). We used AMP4 Alt4 to detect triplex Drd2, Fos, and Drd1, in far red, green, and red channels, respectively. Finally, we incubated sections for 20 s with DAPI. We washed the slides with one washing buffer two times in between incubations. After air drying the slides, we coverslipped them with a Fluoroshield mounting medium (Sigma-Aldrich).

### Statistical analysis

Data were analyzed using GraphPad Prism (GraphPad Software). Two-tailed, unpaired Student’s *t* test or two-way ANOVA with Sidak's post-tests were used to analyze the primary data, except for locomotor and automated stereotypy curve data, which were analyzed using nonlinear curve-fit analysis. Nonlinear regression was chosen over repeated measures (RM) ANOVA for analyzing the time series data because of (1) the nonlinearity of the dependent variables; (2) changes in the treatment effects over time; and (3) the ordering of time points is ignored in RM ANOVA calculations. Comparisons of the slopes of the regressions were analyzed using unpaired *t* tests. Specific statistical analyses for each data set are described in the results and figure legends. All data are reported as the mean ± standard error of the mean. Geisser–Greenhouse corrections were used for ANOVA where appropriate. Pearson’s correlations were used for the analysis of cFos and behavioral data. Schematics were created with BioRender.com.

## Results

### EAAT3 is reversibly and selectively expressed in the forebrain of *Slc1a1*-OE mice

To examine the effect of *Slc1a1* overexpression on behavior and neural activity in mice, we generated *Slc1a1*-OE mice using the Flexible Accelerated STOP-tetO system (Tanaka et al., 2010). *Slc1a1*-OE mice were bred by first crossing *Slc1a1*-tetO-STOP mice with *Pgk1*-flpo mice to excise the Neo-STOP construct (Fig. 1*A*). The resulting progeny were then crossed with *CaMKII*-tTA mice to overexpress *Slc1a1* selectively in forebrain neurons in a doxycycline (dox)-dependent manner. To validate this system, we measured striatal EAAT3 protein expression via Western blot in *Slc1a1*-tetO*/CamKII*-tTA mice fed five different doses of dox-chow (Fig. 1*B*,*C*). As expected, we saw a dose-dependent effect of dox on EAAT3 expression (one-way ANOVA, *F*_(7,16)_ = 53.81, *p *< 0.001). We chose 50 mg/kg of dox-chow as our model of overexpression since this is closer to the expression levels seen in humans with the OCD-associated allele (Wendland et al., 2009; Veenstra-VanderWeele et al., 2012) compared with the supraphysiological expression seen in the 0 mg/kg group. *Slc1a1*-OE mice fed 40 mg/kg of dox-chow showed increased EAAT3 expression in the cortex, striatum, and hippocampus, but not in the midbrain (two-way ANOVA; region–genotype interaction, *F*_(3, 24)_ = 7.108, *p *= 0.0014; region, *F*_(3, 24)_ = 7.108, *p *= 0.0014, genotype, *F*_(1, 24)_ = 58.26, *p *< 0.0001; Sidak's multiple comparisons: cortex, *p = *0.0032; striatum, *p < *0.0001; hippocampus, *p = *0.026; and midbrain, *p = *0.7704; Fig. 1*D*).

### *Slc1a1*-OE mice show no baseline changes in anxiety-like or repetitive behaviors but show increased behavioral response to amphetamine

**Figure 2.**
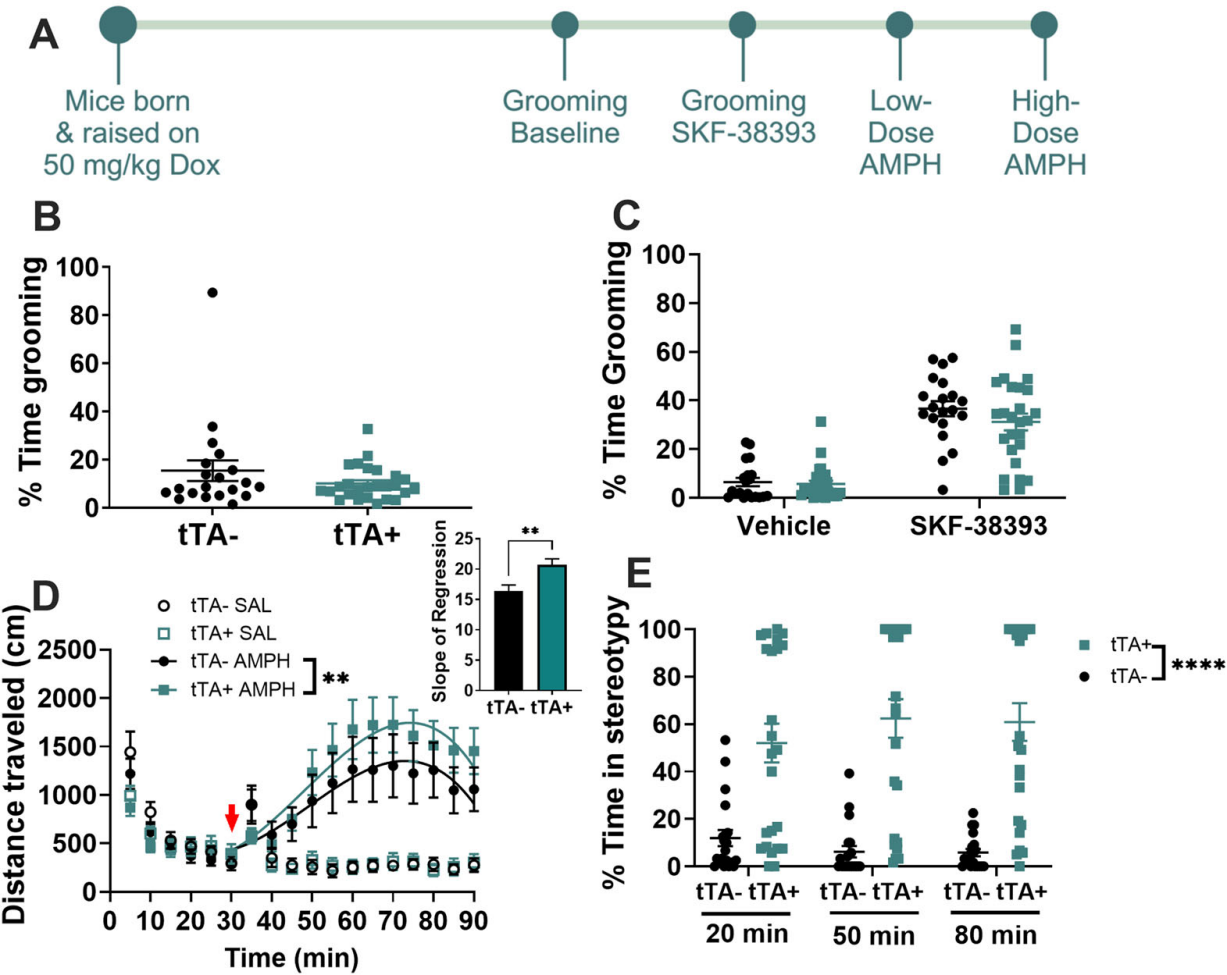
Lifetime *Slc1a1*-OE mice show no differences in baseline or induced grooming but show potentiated behavioral response to both low-dose and high-dose amphetamine. ***A***, Schematic showing experimental timeline. There were no significant differences in the % time grooming between *Slc1a1*-OE (tTA+) mice and tTA− controls either at (***B***) baseline or (***C***) following the administration of SKF-38393. ***D***, *Slc1a1*-OE mice (tTA+) show significantly higher levels of locomotion following amphetamine administration (3.0 mg/kg) relative to tTA− controls (curve-fit analysis, *F*_(4, 802)_ = 3.61, **p < 0.01; slope of regression, unpaired *t* test, *t*_(43)_ = 2.986, **p < 0.0046). The red arrow indicates amphetamine injection at *t* = 30. ***E***, *Slc1a1*-OE (tTA+) mice also show significantly higher levels of stereotypy relative to tTA− controls following a high dose (8.0 mg/kg) of amphetamine (RM ANOVA, main effect of genotype; *F*_(1, 43)_ = 39.16, ****p < 0.0001). See Extended Data Figures 2-1 and 2-2 for more details.

*Slc1a1*-OE mice showed no differences in baseline anxiety-like behaviors. Lifetime *Slc1a1*-OE mice showed no difference in the % time spent in the light side of the light/dark box (one-way *t* test, *t*_(1,36)_ = 0.1687, *p *= 0.87; Extended Data Fig. 2-1*A*) or in the % time spent in the center of the open field (one-way *t* test, *t*_(1, 36)_ = 1.320, *p *= 0.20; Extended Data Fig. 2-1*C*). *Slc1a1*-OE mice additionally showed no differences in OCD-associated grooming behavior, either for baseline or drug-induced grooming (Fig. 2*A*). Lifetime *Slc1a1*-OE mice showed no significant differences in % time spent grooming relative to tTA− controls either at baseline (one-way *t* test, *p *= 0.20 *t*_(1, 44)_ = 1.31; Fig. 2*B*) or following the administration of the D1-agonist SKF-38393 (two-way ANOVA, main effect of drug, *F*_(1, 44)_ = 106.1, *p *< 0.0001; main effect of genotype *F*_(1, 44)_ = 1.52, *p *= 0.22; Fig. 2*C*). However, *Slc1a1*-OE mice showed potentiated behavioral response to amphetamine. Following a low dose of amphetamine (3.0 mg/kg), lifetime *Slc1a1*-OE mice showed significantly more hyperlocomotion relative to tTA− controls (curve-fit analysis, *F*_(4, 802)_ = 3.61, *p *= 0.0063; slope of regression, unpaired *t* test, *t*_(43)_ = 2.986, *p *= 0.0046; Fig. 2*D*). Similarly, lifetime *Slc1a1*-OE mice showed markedly more stereotypy relative to tTA− controls following a high dose (8.0 mg/kg) of amphetamine (RM ANOVA, main effect of genotype; *F*_(1, 43)_ = 39.16, *p *< 0.0001; Fig. 2*E*).

10.1523/ENEURO.0090-24.2024.f2-1 Figure 2-1 **Slc1a1-OE mice show no differences in baseline anxiety-like behavior.** There were no significant differences between *Slc1a1*-OE mice and tTA- controls in anxiety-like behavior. There was no difference in the % time spent in the light side of the light dark test for either lifetime (A) or adult-specific (B) *Slc1a1*-OE mice, and no difference in the % time spent in the center of the open field for lifetime (C) or adult-specific *Slc1a1*-OE mice (D) relative to tTA- controls. Download Figure 2-1, TIF file.

10.1523/ENEURO.0090-24.2024.f2-2 Figure 2-2 **Adult-specific *Slc1a1*-OE mice show no differences in baseline or induced grooming, but show potentiated behavioral response to both low-dose and high-dose amphetamine.** There were no significant differences in the percent time grooming between *Slc1a1*-OE (tTA+) mice and tTA- controls either at (A) baseline or (B) following the administration of SKF-38393. (C) Slc1a1-OE mice (tTA+) show significantly higher levels of locomotion following amphetamine administration (3.0 mg/kg) relative to tTA- controls (Curve-fit analysis, F(4, 676) = 3.72, **p < 0.01; slope of regression, unpaired t test, t (36) = 3.373, **p = 0.0018). Red arrow indicates amphetamine injection at t = 30. Similarly, adult-specific Slc1a1-OE (tTA+) mice showed significantly higher levels of stereotypy relative to tTA- controls following a high dose (8.0 mg/kg) of amphetamine (D, Repeated measures ANOVA, main effect of genotype, F(1, 36) = 14.96, ****p < 0.001). Download Figure 2-2, TIF file.

In prior work, we found that overexpression of *Slc1a1* in dopaminergic neurons during early life leads to heightened amphetamine response, whereas adult overexpression has no impact, pointing to a developmental role for EAAT3 (Chohan et al., 2022). To test the potential impact of forebrain EAAT3 overexpression during development, we separately evaluated mice with EAAT3 overexpression only during adulthood. These adult-specific *Slc1a1*-OE mice displayed no difference in the % time spent in the light side of the light/dark box (one-way *t* test, *t*_(1, 34)_ = 0.7183, *p *= 0.48; Extended Data Fig. 2-1*B*) or in the % time spent in the center of the open field (one-way *t* test, *t*_(1, 34)_ = 0.6386, *p *= 0.53; Extended Data Fig. 2-1*D*) relative to tTA− controls. Additionally, adult-specific *Slc1a1*-OE mice showed no significant differences in % time spent grooming relative to tTA− controls either at baseline (one-way *t* test, *t*_(1, 36)_ = 1.481, *p *= 0.15; Extended Data Fig. 2-2*A*) or following the administration of the D1-agonist SKF-38393 (two-way ANOVA, main effect of drug, *F*_(1, 36)_ = 14.05, *p *< 0.001; main effect of genotype *F*_(1, 36)_ = 0.9508, *p *= 0.34; Extended Data Fig. 2-2*B*). Similar to lifetime overexpressing mice, the adult-specific overexpression cohort showed potentiated hyperlocomotion following a low dose of amphetamine (curve-fit analysis, *F*_(4, 676)_ = 3.72, *p *= 0.0052; slope of regression, unpaired *t* test, *t*_(36)_ = 3.373, *p *= 0.0018; Extended Data Fig. 2-2*C*) and increased stereotypy relative to tTA− controls following a high dose of amphetamine (RM ANOVA, main effect of genotype; *F*_(1, 36)_ = 14.96, *p *= 0.00044; Extended Data Fig. 2-2*D*).

### *Slc1a1*-OE mice show increased cFos expression in the ventral striatum after amphetamine

**Figure 3.**
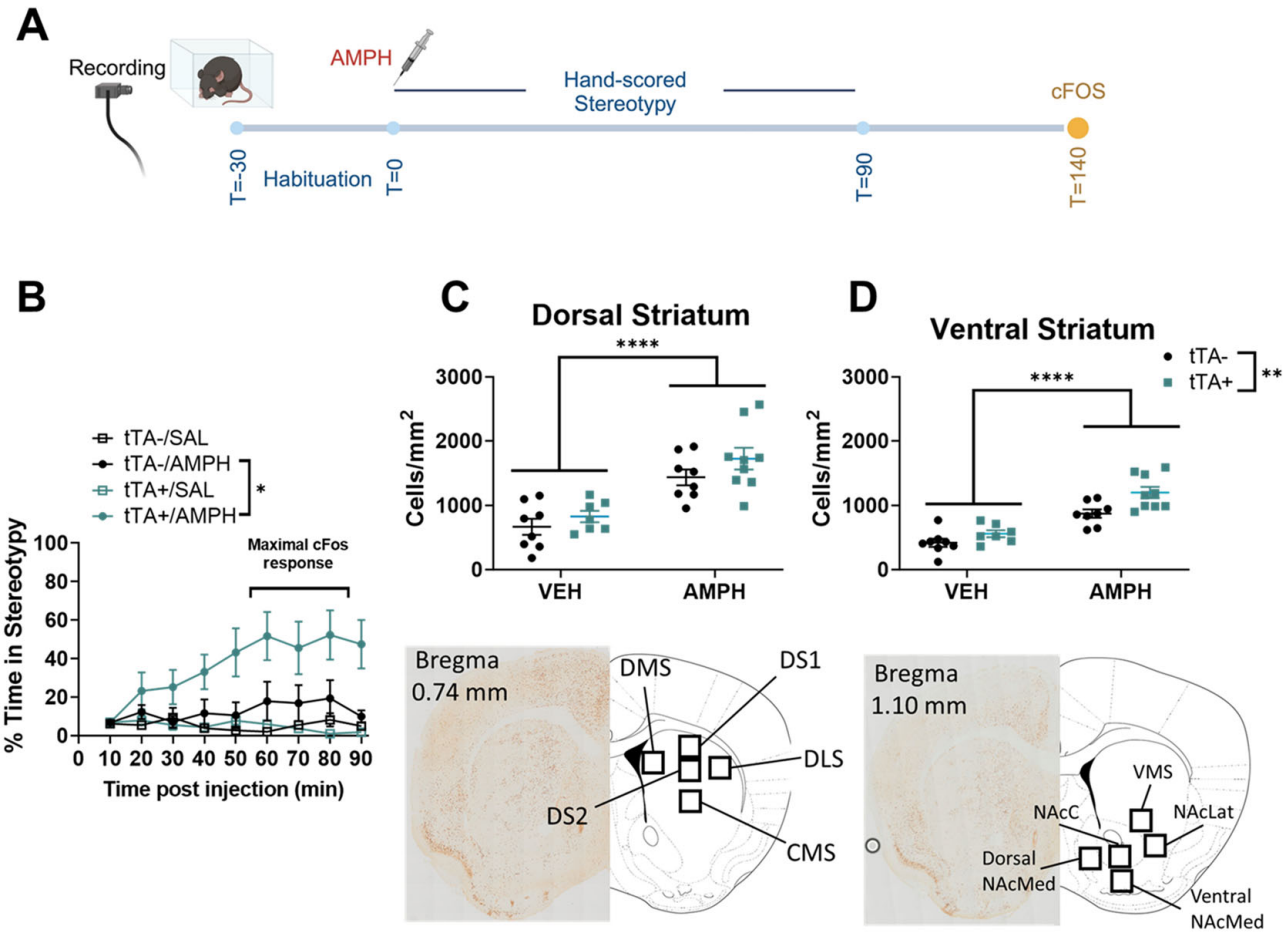
*Slc1a1*-OE mice show increased cFos expression in the VS. ***A***, Schematic showing experimental timeline. ***B***, *Slc1a1*-OE mice show increased stereotypy in response to amphetamine relative to controls (genotype–drug interaction, *p < 0.05). ***C***, Amphetamine increased cFos expression in the DS (pooled data from 5 subregions; drug main effect, ****p < 0.0001). ***D***, Amphetamine increased cFos expression in VS (pooled data from 5 subregions, drug main effect, ****p < 0.0001), and *Slc1a1*-OE mice show increased cFos expression in VS following amphetamine (genotype–drug effect, **p < 0.01). See Extended Data Figure 3-1 for more details.

To investigate the correlates of neural activity underlying the potentiated behavioral response to amphetamine in *Slc1a1*-OE mice, we administered a high dose of amphetamine to a separate cohort of mice and measured striatal expression of the immediate early gene cFos, a marker of neuronal activation (Fig. 3*A*). Similar to the previous cohort, *Slc1a1*-OE mice showed a potentiated stereotypy response to amphetamine, this time scored at nine timepoints postinjection (three-way RM ANOVA, genotype main effect, *F*_(1, 28)_ = 5.35, *p *= 0.028, genotype–drug interaction, *F*_(1,28)_ = 5.79, *p *< 0.024; Fig. 3*B*). Amphetamine significantly increased cFos expression in all 10 striatal subregions spanning the dorsal and ventral striatum (VS) (Extended Data Fig. 3-1; see Extended Data Table 3-1 for statistics). After correcting for multiple comparisons, there was no genotype–drug interaction on the number of cFos-positive cells in any individual subregion (Extended Data Fig. 3-1; Extended Data Table 3-1), although the VMS was closest with a *p* value of 0.019 (corrected *α* = 0.005). We next performed an exploratory analysis by pooling cFos data from the five dorsal subregions into the dorsal striatum (DS) and data from the five ventral subregions into the VS. There was a main effect of drug on cFos expression in the DS (two-way ANOVA, *F*_(1, 28)_ = 37.39, *p *< 0.0001), but no effect of genotype (two-way ANOVA, *F*_(1, 28)_ = 2.728, *p *= 0.11; Fig. 3*C*) and no interaction (two-way ANOVA, *F*_(1, 28)_ = 0.2271, *p *= 0.64). In the VS (Fig. 3*D*), there was a significant effect of both genotype (two-way ANOVA, *F*_(1, 28)_ = 58.68, *p *< 0.0001) and drug (two-way ANOVA, *F*_(1, 28)_ = 10.64, *p *= 0.0029) on cFos expression, but no significant interaction (two-way ANOVA, *F*_(1, 28)_ = 1.692 *p *= 0.204).

10.1523/ENEURO.0090-24.2024.t3-1 Table 3-1 **Table of statistics for two-way repeated measures analysis of cFos in striatal subregions.** Bonferroni corrected α = 0.005, significant *p*-values in bold. NAcLat = lateral nucleus accumbens shell, NAcC = nucleus accumbens core, dorsal NAcMed = dorsal medial nucleus accumbens shell, ventral NAcMed = ventral medial NAc shell, VMS = ventromedial striatum, DLS = dorsolateral striatum, DMS = dorsomedial striatum, DS1 = dorsal striatum-1, DS2 = dorsal striatum-2, CMS = centromedial striatum. Download Table 3-1, XLSX file.

10.1523/ENEURO.0090-24.2024.f3-1 Figure 3-1 **Amphetamine significantly increases cFos in all dorsal and ventral striatum subregions studied.** There was a significant main effect of amphetamine on cFos in all striatal subregions studied. See table 1 for statistics (A) NAcLat = lateral nucleus accumbens shell, (B) NAcC = nucleus accumbens core, (C) dorsal NAcMed = dorsal medial nucleus accumbens shell, (D) ventral NAcMed = ventral medial NAc shell, (E) VMS = ventromedial striatum, (F) DLS = dorsolateral striatum, (G) DMS = dorsomedial striatum, (H) DS1 = dorsal striatum-1, (I) DS2 = dorsal striatum-2, (J) CMS = centromedial striatum. Download Figure 3-1, TIF file.

### An unbiased machine learning classifier shows the potentiated behavioral response in *Slc1a1*-OE mice following amphetamine

**Figure 4.**
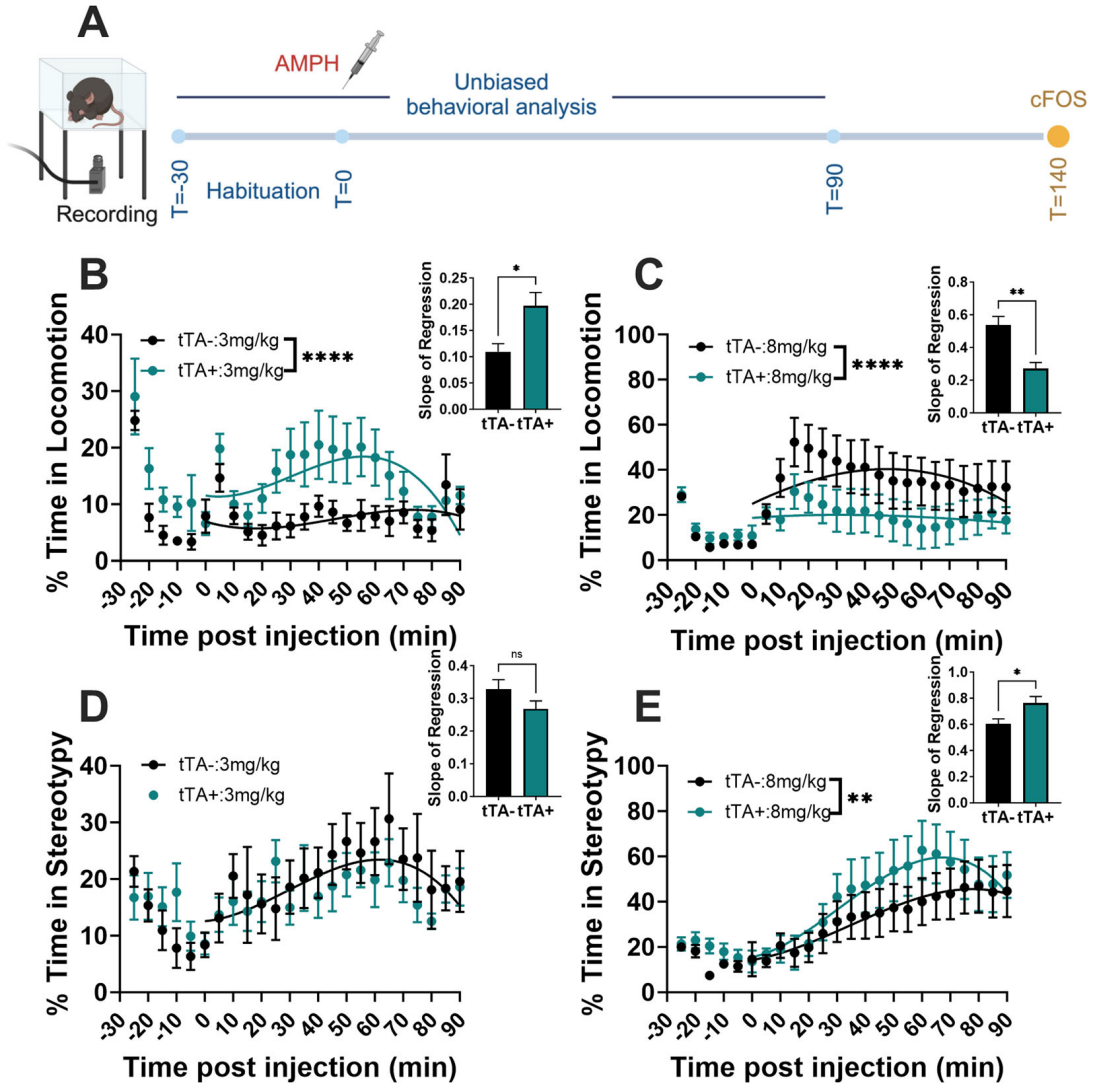
An unbiased machine learning classifier shows the differences in behavior between *Slc1a1*-OE and control mice following amphetamine. ***A***, Schematic showing experimental timeline. ***B***, Following 3.0 mg/kg amphetamine, *Slc1a1*-OE mice show increased % time spent engaged in locomotion relative to tTA− controls (curve-fit analysis, ****p < 0.0001; slope of regression, *p < 0.05). ***C***, Following 8.0 mg/kg amphetamine, *Slc1a1*-OE mice show decreased locomotion relative to tTA− controls (curve-fit analysis, ****p < 0.0001; slope of regression, **p < 0.01). ***D***, Following 3.0 mg/kg amphetamine, *Slc1a1*-OE mice show no differences in stereotypy behavior relative to tTA− controls (curve-fit analysis, ^ns^ *p *= not significant; slope of regression, ^ns^*p *= not significant). ***E***, Following high-dose amphetamine, *Slc1a1*-OE mice spend significantly more time engaged in stereotypy compared with tTA− controls (curve-fit analysis, ** *p *< 0.01; slope of regression, *p < 0.05). See Extended Data Figure 4-1 for more details.

We next sought to obtain a more comprehensive and unbiased assessment of behavior as well as cell-type specific cFos activation patterns in *Slc1a1*-OE mice and tTA− controls following the administration of vehicle, low-dose amphetamine (3.0 mg/kg), or high-dose amphetamine (8.0 mg/kg) (Fig. 4*A*). Behavior in this cohort was analyzed using an unbiased machine learning classifier, and cFos levels were measured in D1- and D2-expressing SPNs using RNAScope (see Materials and Methods). Our classification algorithm revealed six distinct clusters of behavior, corresponding to stereotypy, rest/quiescence, locomotion, stretch–attend posture, sniffing, and grooming (Extended Data video 1). Similar to hand-scored behavior in previous cohorts, *Slc1a1*-OE mice spent significantly more time engaged in locomotion following low-dose amphetamine (nonlinear curve-fit analysis, *F*_(4, 258)_ = 13.04, *p *< 0.0001: slope of regression, unpaired *t* test, *t*_(12)_ = 2.912, *p *= 0.0130; Fig. 4*B*). There was no significant difference between groups in the % time spent in stereotypy following the low dose of amphetamine (nonlinear curve-fit analysis, *F*_(4, 258)_ = 1.60, *p *= 0.18: slope of regression, unpaired *t* test, *t*_(12)_ = 1.623, *p *= 0.1305; Fig. 4*D*). Following high-dose amphetamine, *Slc1a1*-OE mice spent significantly less time engaged in locomotion (nonlinear curve-fit analysis, *F*_(4, 258)_ = 8.63, *p *< 0.0001; slope of regression, unpaired *t* test, *t*_(12)_ = 4.193, *p *= 0.0012; Fig. 4*C*) and significantly more time engaged in stereotypy (nonlinear curve-fit analysis, *F*_(4, 258)_ = 3.07, *p *= 0.017; slope of regression, unpaired *t* test, *t*_(12)_ = 2.515, *p *= 0.0272; Fig. 4*E*). A comprehensive examination of the machine-scored behavior is shown in Extended Data Figure 4-1. Following vehicle treatment, mice engage in primarily quiescence behavior, with no difference between *Slc1a1*-OE mice and controls in % time spent engaging in any of the behaviors identified. In contrast, with amphetamine administration, the patterns of behavior displayed by *Slc1a1*-OE mice differ from controls, with *Slc1a1*-OE mice engaging in more hyperlocomotion following low-dose and stereotypy following high-dose amphetamine.

10.1523/ENEURO.0090-24.2024.f4-1 Figure 4-1 **Traces of B-SOID scored behavior, separated by genotype and drug treatment.** Traces showing individual mouse and average mouse trace for % of total time in stereotypy (A,B), locomotion (C,D), quiescence (E,F), grooming (G,H), exploration (I,J), and sniffing (K,L). Data was analyzed in 5 minute bins. Injection of amphetamine or vehicle occurred at 30 minutes. Download Figure 4-1, TIF file.

10.1523/ENEURO.0090-24.2024.video1 Video 1 Stereotypy Examples. Download Video 1, MP4 file.

### *Slc1a1*-OE mice show increased D1 cFos expression in the VMS following high-dose amphetamine

**Figure 5.**
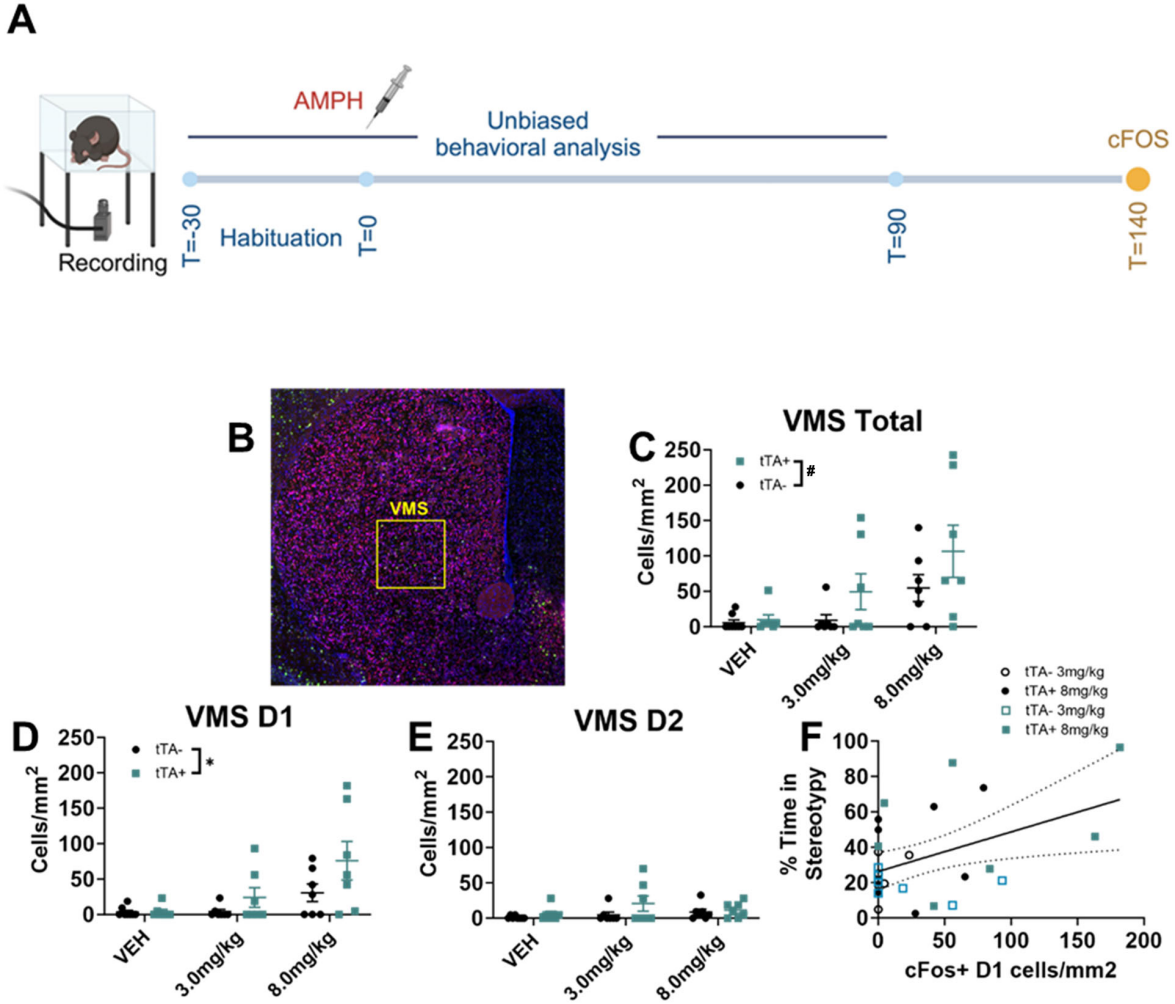
*Slc1a1*-OE mice have increased cFos in D1 neurons following high-dose amphetamine. ***A***, Schematic showing the experimental timeline. ***B***, Representative section showing the fluorescent staining for D1 (red), D2 (purple), cFos (green), and DAPI (blue) in the VMS. ***C***, Amphetamine increases total cFos expression in VMS (drug main effect, *F*_(2, 31)_ = 8.29, **p < 0.01, trend toward a main effect of genotype *F*_(1, 31)_ = 4.010, *^#^p *= 0.054). ***D***, There was a significant main effect of amphetamine (two-way ANOVA, *F*_(2, 31)_ = 9.02, ***p < 0.001) and a main effect of genotype (two-way ANOVA, *F*_(1, 31)_ = 4.35, *p < 0.05) on cFos expression in D1 neurons in the VM. ***E***, There was no effect of drug or genotype or an interaction effect on cFos expression in D2 neurons in the VMS. ***F***, There was a significant positive correlation between the % time spent in stereotypy and VMS D1 cFos expression in mice receiving amphetamine (*r *= 0.44, *p < 0.05).

We next examined cFos expression in D1- and D2-expressing neurons in striatal subregions in mice that were subjected to unbiased machine learning analysis (Fig. 5*A*,*B*). Based on the results from the previous cFos experiment, we focused on the VMS. We found a significant main effect of amphetamine on the overall number of cFos-positive cells in the VMS (drug main effect, *F*_(2, 31)_ = 8.291, *p *= 0.0013; Fig. 5*C*), a trend toward a main effect of genotype (*F*_(1, 31)_ = 4.010, *p *= 0.054), and no significant interaction (drug–genotype interaction *F*_(2, 31)_ = 0.7838, *p *= 0.47). Furthermore, there was a significant main effect of amphetamine on the number of cFos-positive D1 neurons in the VMS (two-way ANOVA, *F*_(2, 31)_ = 9.02, *p *= 0.00082; Fig. 5*D*), as well as a main effect of genotype (two-way ANOVA, *F*_(1, 31)_ = 4.35, *p *= 0.045) on the number of cFos-positive D1 neurons in the VMS, but no interaction (*F*_(2, 31) _= 1.575, *p *= 0.22). There was no effect of drug or genotype or an interaction effect on D2 expression in the VMS (Fig. 5*E*). When looking at all mice receiving amphetamine, there was a significant positive correlation between the % time in stereotypy and VMS D1 neuron cFos expression (*r *= 0.48, *p *= 0.018; Fig. 5*F*).

## Discussion

*SLC1A1* is a candidate gene for OCD, and several polymorphisms in this gene have been associated with the disorder, although none of them have been identified as significant in GWAS studies to date (Stewart et al., 2013; Mattheisen et al., 2015). The rs301430 polymorphism, which has been replicated in multiple association studies of OCD, increases the expression of *SLC1A1* in lymphoblastoid cells, human brain tissue, and a luciferase reporter assay and increases the expression and activity of EAAT3, its protein product (Wendland et al., 2009; Veenstra-VanderWeele et al., 2012). In this study, we modeled this change in EAAT3 expression in mice by overexpressing *Slc1a1* in the striatum, a brain region that has been implicated in OCD in humans and OCD-relevant behaviors in mice (Arnold et al., 2006; Dickel et al., 2006; Stewart et al., 2007; Shugart et al., 2009; Wendland et al., 2009; Samuels et al., 2011; Veenstra-VanderWeele et al., 2012; Cai et al., 2013; Zike et al., 2017; Delgado-Acevedo et al., 2019; Chohan et al., 2022). We found that overexpression of *Slc1a1* had no effect on behavior at baseline but resulted in increased amphetamine-induced repetitive behavior. This behavior was associated with increased cFos expression in the VMS, with a specific correlation with D1 cells in this region. Lastly, we used a novel unbiased machine learning algorithm to cluster behavior following amphetamine and found distinct patterns of amphetamine-induced behavior in *Slc1a1*-OE mice.

Relative to control mice, *Slc1a1*-OE mice showed increased hyperlocomotion following a low dose of amphetamine and increased stereotypy following a high dose of amphetamine. While amphetamine-induced behaviors do not model the symptoms of any particular psychiatric disorder per se, these abnormal repetitive behaviors interfere with adaptive goal-directed behaviors (Wolgin, 2012) and may share some of the underlying neural mechanisms of OCD and OCD family disorders (e.g., tic disorders). Amphetamine has been reported to induce or worsen OCD symptoms in a subset of patients, and dopamine antagonists are used as an adjuvant with SRIs for the treatment of OCD (Denys et al., 2004; Thamby and Jaisoorya, 2019). OCD may represent a hyperdopaminergic state (Denys et al., 2004; Pagliaccio et al., 2023), with observed decreases in D1 and D2 receptor binding in the striatum that could be compensatory (Hesse et al., 2005; Nikolaus et al., 2010; Klanker et al., 2013). Dopamine has also been implicated in other disorders of repetitive behavior, such as tic disorders, with studies showing increased dopamine in patients with Tourette's syndrome using PET (Ernst et al., 1999; Singer et al., 2002), as well as with previous studies using amphetamine challenge to induce stereotypy in a genetic mouse model of Tourette syndrome (Baldan et al., 2014). Interestingly, although amphetamine-induced stereotypies appear to be inflexible, animals can learn to override these stereotypies, indicating that the urge to perform amphetamine-induced behaviors might share some overlap with compulsions in OCD and OCD spectrum disorders (Wolgin, 2012).

In addition to differences in amphetamine-induced behaviors, we also found alterations in striatal activity in *Slc1a1*-OE mice. Following amphetamine administration, *Slc1a1*-OE mice showed increased cFos in VMS, which was accounted for by increased cFos in D1 neurons within this subregion. It is unclear if these differences are secondary to input onto D1-SPNs or due to differences in the intrinsic properties of these neurons. Previous studies in EAAT3-OE mice have shown both pre- and postsynaptic changes to the dopaminergic system, including increased dopamine release and increased D2 receptor expression in the striatum (Escobar et al., 2021), although the mice in these studies were generated using a different method (crossing *CaMKIIα*-Cre with an *Slc1a1*-transgene) and so likely have slightly regional patterns of overexpression (see below for a discussion of behavioral differences). We also found a significant correlation between cFos expression in D1-SPNs and stereotypy behavior in all mice receiving amphetamine. Future studies using larger samples will be needed to further elucidate the relationship between neural activity and behavior, by using cell-type specific manipulations to test whether increased activation of VMS D1-SPNs is responsible for the behavioral phenotypes observed in *Slc1a1*-OE mice.

We found no consistent differences in grooming behavior or anxiety-like behavior between *Slc1a1*-OE mice and controls. The lack of any differences in hand-scored grooming behavior is reinforced by the lack of differences in grooming in the machine-scored behavior. The grooming behavior identified by our B-SOiD clustering algorithm grouped face grooming, body grooming, and scratching as one behavioral cluster. The lack of separate behavioral clusters for each type of grooming is in contrast to other papers utilizing B-SOiD (Hsu and Yttri, 2021) and may be due to relatively sparse body grooming and scratching in these mice. The lack of differences in baseline OCD-relevant behavior in these mice is in contrast to the results of a recently published paper that shows significantly increased time spent grooming and enhanced anxiety-like behavior in a different line of *Slc1a1*-OE mice (Delgado-Acevedo et al., 2019). These differences could be due to several factors, including differences in mouse background, testing conditions, or functional levels of EAAT3 overexpression. Furthermore, while we previously reported no anxiety-like behavioral differences in mice lacking *Slc1a1* (Zike et al., 2017), two previous studies reported increased anxiety-like behavior in *Slc1a1*-KO mice, with one of these studies reporting an increased number of grooming bouts in *Slc1a1*-KO mice as well (Afshari et al., 2017; Bellini et al., 2018). More research is clearly needed to clarify the role of EAAT3 on baseline OCD-relevant grooming and anxiety-like behavior, including the impact of distribution and mechanism of EAAT3 manipulations, mouse strain, and testing conditions.

To the best of our knowledge, this is the first study to examine amphetamine-induced behavior using an unbiased machine learning approach. Previous reports of amphetamine-induced behavior have relied on hand-scoring of stereotypy and related behaviors or locomotor chambers for measuring distance traveled (Kelley, 2001). There are significant weaknesses to these approaches and significant advantages to an unbiased approach. Practically speaking, human scorers are subject to fatigue as well as experimenter bias. In lengthy behavioral testing sessions, it is usually necessary to score only a subset of the session, and it is difficult to score more than one behavior accurately in real time. Furthermore, experimenters must decide which behavior(s) of interest to score a priori, necessarily neglecting other potential changes in behavioral patterns. Neural activity may not neatly correlate with pre-existing categories of behavior, and the unbiased approach has the potential to give us new insight into the neural circuits underlying complex drug-induced behavior. Our data-driven approach identified a behavioral cluster that corresponds to what we identify as stereotypy, and we showed that *Slc1a1*-OE mice engage in more of this behavior than control mice following amphetamine administration.

Our findings also have important limitations. Mice in the regional overexpression study (Fig. 1*D*) were fed 40 mg/kg doxycycline chow, which does not match the dose of doxycycline chow used in other experiments (50 mg/kg). This experiment was conducted after the initial studies and 50 mg/kg dox-chow was unavailable at the time of this experiment; 40 mg/kg was the closest dose available. These cohorts of mice might therefore have different regional levels of EAAT3 overexpression. Our use of RNAscope for cFos colocalization with D1 and D2 receptor expression did not allow us to examine the in vivo temporal dynamics of these two neuronal populations during behavior. Causal experiments and in vivo recording methods would be necessary to test whether VMS D1 cells indeed drive this stereotypic behavior and to understand more broadly what role different cell populations play in amphetamine-induced behavior. Additionally, our selection of a titrated *CaMKII*-tTA approach for overexpression of *Slc1a1* does not easily reconcile with prior reports that used *CaMKII*-Cre to excise a reverse-floxed EGFP cassette to permit *Slc1a1* overexpression using a CMV promoter in the hippocampus, striatum, and cortex (Delgado-Acevedo et al., 2019). Finally, prior findings from studies using *CaMKII*-tTA mice suggest that the *CaMKII* promoter drives transgene expression in both D1- and D2-MSNs (Cazorla et al., 2012; Klug et al., 2012). Combined with a recent report showing indistinguishable expression of EAAT3 in D1- and D2-MSNs (Petroccione et al., 2023), we expect to find similar levels of overexpression in D1- and D2-MSNs in *Slc1a1*-OE mice. However, this was not empirically tested in our mouse model.

In summary, we harnessed a novel, unbiased behavioral approach to demonstrate increased locomotor and stereotyped behavior following amphetamine in mice with striatal overexpression of *Slc1a1*. This increased amphetamine-induced behavior was associated with increased cFos expression in VMS neurons in *Slc1a1*-OE mice, with a specific correlation of stereotypy with cFos expression in D1 neurons within this region. We also found a significant increase in D1 neuron activation in *Slc1a1*-OE mice following a high dose of amphetamine, indicating that this cell population may be particularly important for the behavioral effects of amphetamine in these mice. In future work, we believe that an unbiased approach to behavioral analysis, as implemented here, when combined with in vivo recording technologies, could be a particularly valuable approach to further understanding amphetamine-induced behavior.

## Synthesis

Reviewing Editor: Jibran Khokhar, Western University Department of Anatomy and Cell Biology Decisions are customarily a result of the Reviewing Editor and the peer reviewers coming together and discussing their recommendations until a consensus is reached. When revisions are invited, a fact-based synthesis statement explaining their decision and outlining what is needed to prepare a revision will be listed below. The following reviewer(s) agreed to reveal their identity: NONE. Note: If this manuscript was transferred from JNeurosci and a decision was made to accept the manuscript without peer review, a brief statement to this effect will instead be what is listed below.

This is a wonderfully formulated study with important findings related to the commonly associated OCD gene Slc1a1/EAAT3. The authors have done a great job of addressing the reviewer comments.
