# Supplementary figures and images for: Forebrain EAAT3 Overexpression Increases Susceptibility to Amphetamine-Induced Repetitive Behaviors
Source: eNeuro. 2024 Apr 8;11(4):ENEURO.0090-24.2024. doi: 10.1523/ENEURO.0090-24.2024 (PMC11012153; doi:10.1523/ENEURO.0090-24.2024)

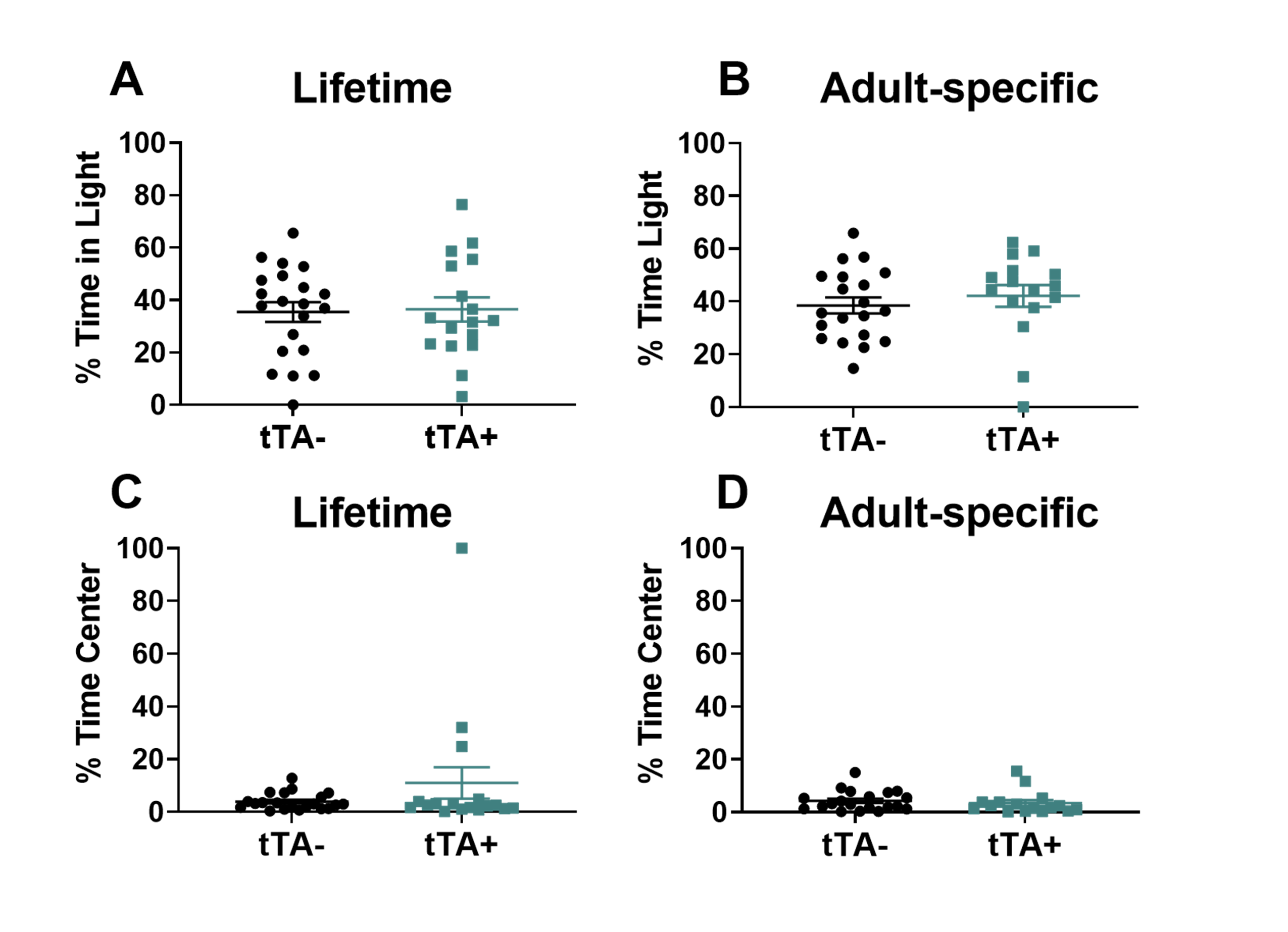

Supplement: Figure 2-1 — Slc1a1-OE mice show no differences in baseline anxiety-like behavior. There were no significant differences between Slc1a1-OE mice and tTA- controls in anxiety-like behavior. There was no difference in the % time spent in the light side of the light dark test for either lifetime (A) or adult-specific (B) Slc1a1-OE mice, and no difference in the % time spent in the center of the open field for lifetime (C) or adult-specific Slc1a1-OE mice (D) relative to tTA- controls. Download Figure 2-1, TIF file. [file eneuro-11-ENEURO.0090-24.2024-s002.tif]

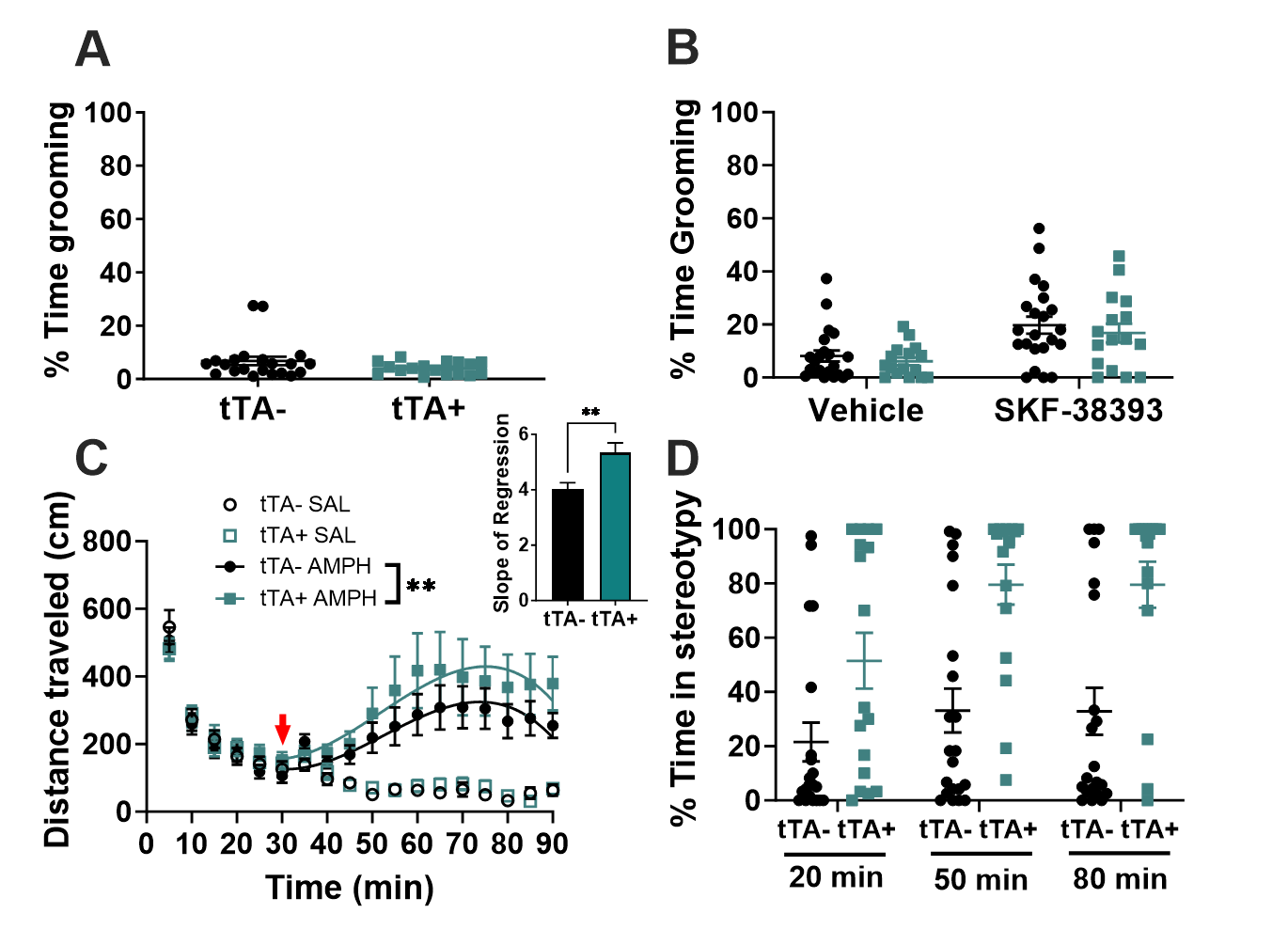

Supplement: Figure 2-2 — Adult-specific Slc1a1-OE mice show no differences in baseline or induced grooming, but show potentiated behavioral response to both low-dose and high-dose amphetamine. There were no significant differences in the percent time grooming between Slc1a1-OE (tTA+) mice and tTA- controls either at (A) baseline or (B) following the administration of SKF-38393. (C) Slc1a1-OE mice (tTA+) show significantly higher levels of locomotion following amphetamine administration (3.0 mg/kg) relative to tTA- controls (Curve-fit analysis, F(4, 676) = 3.72, **p < 0.01; slope of regression, unpaired t test, t (36) = 3.373, **p = 0.0018). Red arrow indicates amphetamine injection at t = 30. Similarly, adult-specific Slc1a1-OE (tTA+) mice showed significantly higher levels of stereotypy relative to tTA- controls following a high dose (8.0 mg/kg) of amphetamine (D, Repeated measures ANOVA, main effect of genotype, F(1, 36) = 14.96, ****p < 0.001). Download Figure 2-2, TIF file. [file eneuro-11-ENEURO.0090-24.2024-s003.tif]

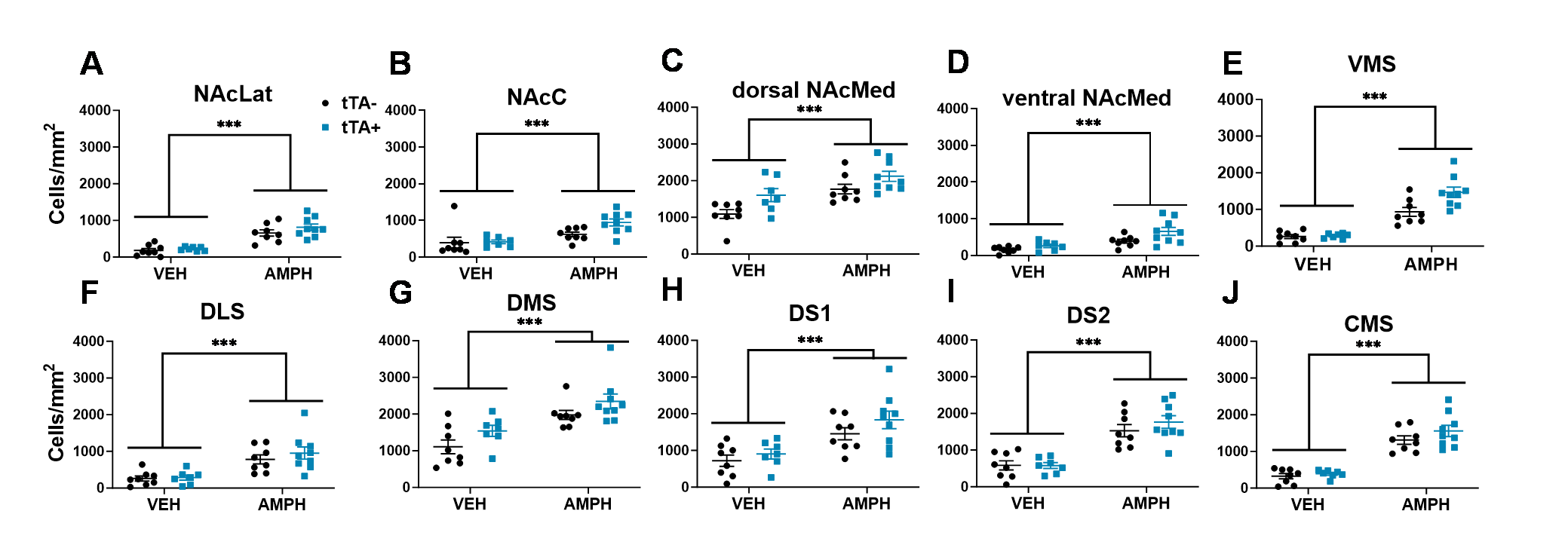

Supplement: Figure 3-1 — Amphetamine significantly increases cFos in all dorsal and ventral striatum subregions studied. There was a significant main effect of amphetamine on cFos in all striatal subregions studied. See table 1 for statistics (A) NAcLat = lateral nucleus accumbens shell, (B) NAcC = nucleus accumbens core, (C) dorsal NAcMed = dorsal medial nucleus accumbens shell, (D) ventral NAcMed = ventral medial NAc shell, (E) VMS = ventromedial striatum, (F) DLS = dorsolateral striatum, (G) DMS = dorsomedial striatum, (H) DS1 = dorsal striatum-1, (I) DS2 = dorsal striatum-2, (J) CMS = centromedial striatum. Download Figure 3-1, TIF file. [file eneuro-11-ENEURO.0090-24.2024-s004.tif]

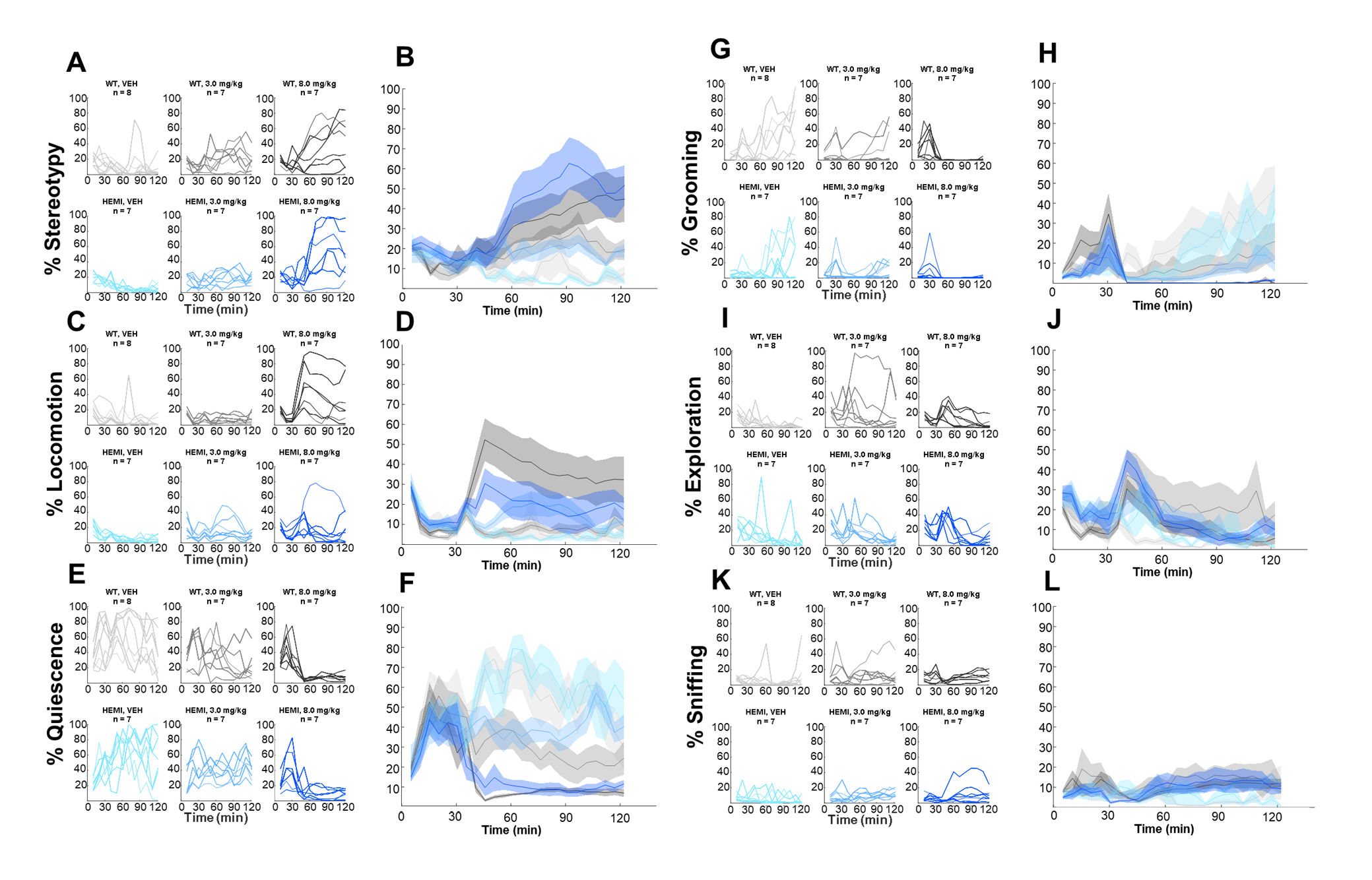

Supplement: Figure 4-1 — Traces of B-SOID scored behavior, separated by genotype and drug treatment. Traces showing individual mouse and average mouse trace for % of total time in stereotypy (A,B), locomotion (C,D), quiescence (E,F), grooming (G,H), exploration (I,J), and sniffing (K,L). Data was analyzed in 5 minute bins. Injection of amphetamine or vehicle occurred at 30 minutes. Download Figure 4-1, TIF file. [file eneuro-11-ENEURO.0090-24.2024-s005.tif]
